# A tri-component knee plug for the 3rd generation of autologous chondrocyte implantation

**DOI:** 10.1038/s41598-020-73863-x

**Published:** 2020-10-12

**Authors:** Lobat Tayebi, Zhanfeng Cui, Hua Ye

**Affiliations:** 1 Institute of Biomedical Engineering, Department of Engineering Science, University of Oxford, Oxford, OX3 7DQ UK; 2 Marquette University School of Dentistry, Milwaukee, WI 53233 USA

**Keywords:** Biomaterials, Biomedical engineering

## Abstract

Here, we report a newly designed knee plug to be used in the 3rd generation of Autologous Chondrocyte Implantation (ACI) in order to heal the damaged knee cartilage. It is composed of three components: The first component (Bone Portion) is a 3D printed hard scaffold with large pores (~ 850 µm), made by hydroxyapatite and β-tricalcium phosphate to accommodate the bony parts underneath the knee cartilage. It is a cylinder with a diameter of 20 mm and height of 7.5 mm, with a slight dome shape on top. The plug also comprises a Cartilage Portion (component 2) which is a 3D printed gelatin/elastin/sodium-hyaluronate soft thick porous membrane with large pores to accommodate chondrocytes. Cartilage Portion is secured on top of the Bone Portion using mechanical interlocking by designing specific knobs in the 3D printed construct of the Cartilage Portion. The third component of the plug (Film) is a stitchable permeable membrane consisting of polycaprolactone (PCL) on top of the Cartilage Portion to facilitate sliding of the knee joint and to hold the entire plug in place while allowing nutrients delivery to the Cartilage Portion. The PCL Film is prepared using a combination of film casting and sacrificial material leaching with a pore size of 10 µm. It is surface modified to have specific affinity with the Cartilage Portion. The detailed design criteria and production process of this plug is presented in this report. Full in vitro analyses have been performed, which indicate the compatibility of the different components of the plug relative to their expected functions.

## Introduction

Bone ends within knee joint is covered with articular cartilage, a tissue which is highly prone to injury. While healthy cartilage is smooth, allowing relaxed sliding of the knee joint, damaged cartilage can be rough—exposing the underlying bone^1,2^. Articular cartilage injury in knee joints is a challenging medical problem. A growing number of patients are suffering from symptomatic focal knee cartilage lesions, which can be attributed to the limited self-repairing capacity of articular cartilage^3–7^. Despite development of different medical procedures to provide knee pain relief, there are arguments about the efficacy of current methods in treatment of cartilage defects^8,9^.

The strategies that address knee cartilage problems include:

1. Palliation: Such as arthroscopic chondroplasty and debridement, which are mostly related to minor and moderate cartilage wear. For example, arthroscopic chondroplasty is an outpatient procedure in which the injured tissue is detached to allow healthy cartilage to be developed in its place. This procedure involves using an arthroscope as a specific video camera to help make tiny incisions on the sides of the knee to ultimately repair a small area of damaged cartilage within the knee^10–12^. In the arthroscopic debridement procedure, a washout or joint lavage eliminates debris around the injured cartilage or bone using specific surgical instruments^13–16^.
2. Repair: Such as microfracture drilling, which is an outpatient and minimally-invasive practice, mostly utilized following sports injuries. In this procedure, the surgeon drills small fractures into the bone at the base of the damaged cartilage in the knee joint to repair the injured area by stimulating the growth of healthy "scar" cartilage, called fibrocartilage. Special care at home may be needed for 6–8 weeks after the procedure^17–19^.
3. Restoration: Such as osteochondral autograft or allograft, and autologous chondrocyte implantation (ACI), which are applied for serious cartilage injuries.

Osteochondral autografts or allografts are often used to treat knee cartilage injuries that uncover the underlying bone to replace the construct of cartilage/bone by either the patient’s tissue (osteochondral autograft) or a donor’s body (osteochondral allograft)^20–22^. Osteochondral autograft is a good option, yet limited to small defects due to the limitation of tissue supply from the patient’s body^8,23^. Additionally, osteochondral allograft has the risk for immune rejection^24,25^.

The ACI method, a fairly new and state-of-the-art technique that can be employed for knee cartilage regeneration, is traditionally a two-stage process. In the first-stage, a minor portion of cartilage from the patient’s knee is harvested. The biopsy is then enzymatically treated in the laboratory to extract the chondrocytes (cartilage-producing cells). They are expanded for a few weeks and then returned to the patient’s knee during an open operative procedure that constitutes the second-stage^26–29^.

Among all the above approaches, microfracture and ACI are more popular. However, the formation of fibrocartilage microfracture—instead of natural hyaline cartilage—is problematic, as fibrocartilage is not hard enough and can be deteriorated within a few years^30^. ACI, despite being a more difficult method, replaces the injured cartilage with natural hyaline cartilage, which is a real advantage of this method for severe damages.

ACI has evolved rapidly since its first description in 1994^31^, and has gone through a few generations. In the 1st generation of ACI, cultured chondrocytes were transferred to the injured region as a liquid suspension. Then a strong fibrous tissue, periosteum, was used to cover the area. Due to the use of periosteum, this generation is often called ACI-P, in which ‘P’ refers to periosteum. However, hypertrophy or ossification of the patched periosteum is known to be challenging^32^. Moreover, harvesting periosteum from the patient is a procedure that has its own complexities and usually causes discomfort for the patients^30^. In the 2nd generation of ACI, a collagen cover was employed as a replacement for periosteum. This generation is usually called ACI-C, in which C refers to collagen. In the 2nd generation, cells were still employed as liquid suspensions, and the collagen cover was stitched in the area^30^. Cell retention was a major challenge in the 1st and 2nd generations of ACI, thus a new generation has been developed (3rd generation), in which the cells are delivered, loaded or seeded to the injured area by the use of a 3-dimensional (3D) matrix, including a scaffold or a membrane (such as collagen membranes), known as the matrix-assisted autologous chondrocyte transplantation (MACT)^30,33,34^. Basically, this generation aims to use a 3D object/environment in order to accommodate the cultured chondrocytes to protect the cells, distribute them more homogeneously, facilitate their re-differentiation and aide the surgical implantation^33,35^.

Different scaffolds have been developed for this purpose. Such scaffolds must not only protect the cells, but should also distribute them more homogeneously, help their re-differentiation and make the surgical procedure easier^33,35^. Here, the scaffolds made for tissue engineering applications, especially the osteochondral scaffolds, have become very useful. Three types of biomaterial-based scaffolds have been used for this purpose^36^:

1. Monophasic scaffolds, in which one material with homogenous porosity is used.
2. Biphasic scaffolds, in which either two different materials or one material consisting of two parts with different porosities are used.
3. Triphasic or multiphasic scaffolds, are scaffolds with three or more materials, or one material consisting of three or more parts with different porosities are employed^36^.

Biphasic and multiphasic osteochondral scaffolds are more effective for cartilage regeneration and can be made with both natural and synthetic materials^36–40^. The materials selected for different phases of the osteochondral constructs depend on the fabrication method of the construct^41^. The other factor in material selection is the approach for incorporating the cells into the scaffolds^41^. For example, if the intended approach is encapsulation of cells within the scaffolds during the fabrication, the material selection is different than a scenario in which the cells are seeded into the scaffolds after fabrication^41^.

One of the approaches in making such bi/multiphasic osteochondral scaffolds is fabricating separate bone and cartilage parts, then connecting them together by different methods, such as press fitting, suturing or using a glue^42^. For example, in scaffolds made of two phases of fibrin/polycaprolactone (PCL) or PCL/PCL-tricalcium phosphate, fibrin glue was used to connect the phases after seeding them separately in chondrogenic (for cartilage regeneration) and osteogenic (for developing bone) media^43,44^. There are reports indicating that such methods can result in poor connection between cartilage and bone parts and ultimately lead to dissociation^45^.

In another biphasic scaffold, agarose gel and decellularized bone were used to make the cartilage and bone parts, respectively^44^. Agarose provided the good mechanical property for immature chondrocytes^44,46^, while the decellularized bone was helpful in terms of making osteoinductive construct with mechanical properties and biochemical composition similar to bone^44^. Agarose layer penetrated into the bone portion and solidified to form an interface between the two cartilage and bone segments. However, the ultimate interfacial tissue after incubation in an osteochondral bioreactor was distinctive from the interfaces of a native tissue^44^.

Attempting to make multiphasic osteochondral scaffolds, Harley et al. used sequences of collagen type I/glycosaminoglycan/calcium phosphate (CGCaP) in scaffolds fabricated by employing the freeze-drying technique^47^. In another study by the same research group, they made a specific multiphasic scaffold using a mineralized CGCaP and an unmineralized collagen type II/glycosaminoglycan (CG) suspensions for osteochondral regeneration^48^. In this scaffold, the interface of the two parts were formed by interdiffusion amongst the suspensions of each layer before freeze-drying^48^. The authors did not investigate the cell proliferation in the fabricated scaffolds.

The method of interfusion to form an interface between phases of an scaffolds was also used by Wang et al., where they employed articular cartilage ECM (ACECM) for cartilage regeneration and hydroxyapatite (HA) for bone regeneration in a biphasic osteochondral scaffold^49^. It was evident that the cartilage portion could accommodate the rabbit chondrocytes well, yet not many cells were observed at the interfacial region. The fact that no chondrocytes migrated into the bony portion demonstrated the barrier characteristics of the interface^49^.

A grouping of β-tricalcium phosphate (TCP) blocks and scaffold-free sheet of mesenchymal stem cell (MSCs) was used by Miyagi et al*.* for osteochondral applications^50^. In a similar strategy, centrifuged chondrocyte cell sheets were employed by Niyama et al.^51^. However, it was found that using such cell-sheet method has some technical difficulties and limitations, which are mostly related to the use of one kind of cell culture medium in a cell-sheet construct to stimulate the differentiation of both osteoblasts and chondrocytes^50,51^.

3D printing, in combination with other methods, has also been employed for making appropriate scaffold for this purpose. For example, Tuan et al. fabricated a biphasic scaffold, by combination of FDM 3D-printing method and electrospinning, in which the PCL and PCL/TCP were used for the cartilage and bone parts, respectively^52^. The investigators have implanted this scaffold in pigs and achieved successful cartilage regeneration. However, it was found that not only the scaffold design affected the results, but also the implantation site had considerable influence (e.g., medial condyle against patellar groove)^52^.

In another similar example, A multiphasic scaffold was produced by Jeon et al., in which one part was composed of 2% alginate and the other part was a biphasic scaffold made of PCL^53^ and a combination of FDM 3D-printing method and electrospinning was used to fabricate the construct^53^. The two phases were press-fitted to allow the alginate to infiltrate into the PCL part. The scaffold was implanted in rats, and the histological analysis showed separation of some alginate from PCL, indicating the failure of the interface^53^.

Focusing on 3D printing method and possibility of mechanical interlocking in this technique, we report a new design of a knee plug composed of three components: two components to accommodate the chondrocytes and the bone cells underneath the cartilage, and one component as a membrane that covers the whole construct.

The novelty of this work is in the manufacturing of a ready-to-use, highly reproducible 3D printed construct for ACI 3rd generation surgery of damaged knee cartilage, with controlled pore size and structure. The construct has the size of the area that is typically removed from the knee for a damaged cartilage treatment and is made of stitchable materials that can accommodate suitable cells. Utilizing a 3D printing approach to develop this plug provides the controllability over the pore size, which is important for the growth of new tissues. Detailed fabrication procedures of this knee plug, along with the mechanical properties and in vitro analyses of different components, are presented in this paper. To the best of our knowledge, such a tri-component 3D printed plug for the knee cartilage repair has not yet been developed.

## Materials and methods

### Design

**Figure 1:**
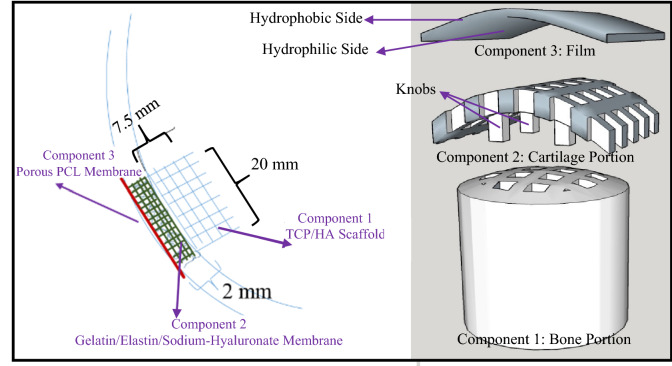
A schematic view of the tri-component knee plug.

As shown in Fig. 1, the designed plug is composed of three separate components that can be fixed either by mechanical interlocking or suturing. The bottom part (Component 1, Bone Portion) is a 3D printed hard scaffold made of TCP and HA that is sintered to yield sufficient mechanical properties. Once positioned within the designated area, bioactive TCP and HA can assist growth of osteoblasts within the bone layer, and growing bone secures the joint plug in place.

This scaffold is basically a porous cylinder with a diameter of 20 mm and height of 7.5 mm, with a slight dome shape on top. The size of pores in this 3D printed construct are approximately 850 µm. It is configured for insertion into the opening of the bone layer.

The plug further includes a 3D printed soft scaffold for accommodating chondrocytes. This middle part, (Component 2, Cartilage Portion) is made of gelatin/elastin/sodium hyaluronate. It has a thickness of 2 mm with the approximate pore size of 892 µm and, as shown in the results, is appropriate for the attachment and growth of chondrocytes.

The top part (Component 3, Film) is a porous membrane made of PCL, which allows nutrients to diffuse, yet prevents cells from migrating out of the construct. It is fabricated in a way that is stitchable/suturable. The pore size of this membrane is approximately 10 µm.

It is favorable to have engagement means to attach different components. Such engagement may occur before implanting the plug into the body. Alternatively, it can happen after inserting the plug. To engage the Cartilage and Bone Portions, some knobs are designed in the 3D printed construct of the Cartilage Portion that can interlock these two portions and confine their locations.

The engagement means between the Film and Cartilage Portion can be in the form of suturing and/or by modifying the surface of the Film so it can have specific affinity to the Cartilage Portion. One effective approach that we follow in this paper, is treating one side of the Film with NaOH. It significantly increases the wettability of the side faced to the Cartilage Portion to facilitate the engagement of the Film to this portion. Hydrophobic surface of the other side of the Film prevents the cells to migrate outside of the plug.

### Materials

TCP, HA, PCL (Mn 80,000), polyethylene glycol (PEG, Mn 2000) and gelatin (Type A, from porcine skin, Gel strength ~ 300 g Bloom) were purchased from Sigma (USA). Carboxymethyle cellulose (CMC), sodium tripolyphosphate (TPP), 1-Ethyl-3-(3-dimethylaminopropyl) carbodiimide (EDC) and *N*-hydroxysuccinimide (NHS) were purchased from Alfa Aesar (USA). Elastin with a molecular weight of 60 kDa (Elastin-Soluble, No. ES12) was purchased from Elastin Products Company, Inc. Sodium Hyaluronate (Research Grade, 500–749 KDa) was obtained from Lifecore Biomedical. All the solvents were of reagent grade.

### 3D printing of the bone and cartilage portions

A printable paste composed of TCP, HA, CMC and TPP was used to print the Bone Portion. To prepare the ink, 12 g TCP, 3 g HA, 0.5 g TPP and 0.075 g were added to 5.75 mL water and homogenized at 2000 rpm for 2 min(min) using a centrifugal mixer (Thinky, USA). Supplementary File 1 (SF 1) explains in detail the reason of selecting this composition as the ink for printing the scaffold of Bone Portion.

Finally, the ink was loaded into standard Nordson cartridges, and the Bone Portion was printed using a 3D-BIOPLOTTER system (EnvisionTEC, Germany) by adjusting the printing parameters. The optimized parameters are presented in Table 1.

**Table 1:** Parameters used in 3D printing of the bone portion of the plug.

Parameter	Value
Dimensions	20 mm diameter × 7.5 mm height with a solid rod in the middle (with diameter of 4 mm)
Cartridge temperature	22 °C
Platform temperature	22 °C
Pressure	2 bars
Nozzle speed	4 mm/s
Distance between strands	2 mm
Slicing	300 µm
nozzle diameter	410 µm

The resulting constructs were allowed to air-dry overnight before being transferred to a furnace. The samples were heated up to 600 °C at the rate of 3 °C/min and held at this temperature for 2 h (h) to eliminate the organic additives. The temperature was then raised to 1100 °C at the rate of 5 °C/min and kept at this temperature for 4 h to ensure complete sintering of the ceramic scaffolds. Imaging of the scaffolds and measuring their surface roughness were performed using a 3D confocal laser scanning microscope (LEXT, Olympus).

To print the Cartilage Portion, a solution of gelatin/elastin/sodium hyaluronate in deionized (DI) water was prepared and used as the ink. The concentration of gelatin, elastin and sodium hyaluronate was adjusted to 8%, 2% and 0.5% w/v, respectively. The ink was loaded into standard Nordson cartridges and the Cartilage Portion was printed using the parameters listed in Table 2.

**Table 2:** Applied parameters to 3D print the cartilage portion of the plug.

Parameter	Value
Dimensions	20 mm diameter × 2 mm height
Cartridge temperature	31 °C
Platform temperature	12 °C
Pressure	2 bars
nozzle speed	15 mm/s
Distance between strands	1.5 mm
Total number of layers	8
Nozzle diameter	410 µm

The printed constructs were then cross-linked using a solution of 6 mg/mL EDC and 0.75 mg/mL NHS in 70% v/v ethanol for 4 h. To remove the residual cross-linker, the constructs were washed carefully through soaking in the large amount of DI water. The prepared constructs were stored in pure ethanol inside a − 20 °C freezer to be used after rehydration.

### Fabrication and surface treatment of the film

Porous PCL Film was prepared using the combination of film casting and sacrificial material leaching methods^54^. Briefly, 1 g PCL was dissolved in 15 mL of 2,2,2-trifluoroethanol, and varying amounts of PEG 2000 g/mol (0, 0.2, 0.4, 1 and 1.5 g) were added to the solution. After complete dissolution, the solution was casted, and the solvent was allowed to evaporate overnight. The obtained membranes were soaked in water to eliminate/sacrifice PEG thereby the porous structures were achieved by sacrificing PEG.

To treat the surface of the Film that faced toward the cells, the prepared membrane was allowed to float on 10% w/v NaOH overnight. The Film was finally rinsed with copious amounts of water to remove residual NaOH.

### Characterization and biological evaluation of the bone portion

#### Mechanical properties

The mechanical properties of the Bone Portion were measured using an Electromechanical Precision Universal Tester (AGS-X 5 kN, Shimadzu, Japan). The samples were tested in compression mode using a 5 kN load cell and crosshead speed of 1 mm/min.

#### Osteoblast attachment and proliferation

Human osteoblasts (HOB, Cell Applications, USA) were cultured under standard aseptic conditions^55^. The cells were cultured in standard flasks and nourished with Dulbecco Modified Eagle Medium (DMEM) supplemented with 10% v/v fetal bovine serum (FBS), 100 U/mL penicillin, 100 µg/mL streptomycin and 0.25 µg/mL amphotericin every 2 days until a confluency of 90% was reached. The cells were trypsinized using TrypLE (Gibco, US) and sub-cultured. The cells of the third passage were used to seed the scaffolds at a density of 2500 cells per mm^2^ of scaffolds (cells/mm^2^).

Cell attachment to the scaffolds was evaluated using scanning electron microscopy (SEM, JEOL-JSM6510, Japan)^55,56^. At certain time intervals (i.e. 3, 7, 14 and 21 days), the constructs were taken out, washed with PBS and fixed using Karnovsky’s fixative (composed of 2% paraformaldehyde; 2.5% glutaraldehyde in 0.1 M phosphate buffer pH 7.4) for 2 h. The samples were then fixed using 1% w/v Osmium Tetroxide solution, dehydrated using ascending ethanol series (30, 50, 75, 95 and 100% v/v) and left overnight to air dry at room temperature. After being sputter-coated with gold, the samples were imaged using back-scattering and secondary electron modes at different magnifications.

The capability of the prepared scaffolds to induce cell proliferation was evaluated using prestoblue assay^57,58^. At certain time intervals (i.e. 3, 7, 14 and 21 days), the cell culture media was replaced with 10% v/v prestoblue reagent (life technologies, USA) in phenol red free DMEM and incubated at 37 °C and 5% CO_2_ for 1.5 h. The fluorescence intensity of the reagent was recorded at the excitation/emission wavelengths of 540/590 nm using a micro-plate reader (Synergy HTX, BioTek, USA)^57^.

### Characterization and biological evaluation of the Cartilage Portion

#### Degradation

Sample degradation rate was measured by monitoring the weight of samples over time. Samples were immersed in phosphate buffered saline (PBS) and kept at 37 °C in a shaker incubator (IKA KS 3000) for 60 days. At certain times, from day 0 to day 60, samples were taken out, weighed and returned to the container. The ratio of the recorded weight to the initial weight at each time point was reported as a function of time.

#### Chondrocyte attachment and proliferation

Normal human chondrocytes (Cell Applications Inc., USA) were cultured and sub-cultured under standard aseptic conditions. At the confluency of 90%, chondrocytes were trypsinized and resuspended in fetal bovine serum (FBS, Sigma-Aldrich, USA) to be seeded to the scaffolds of the Cartilage Portion. Scaffolds were disinfected using 70% ethanol, washed and seeded with a density of 3 × 10^5^ cells/scaffold in 12-well culture plates and allowed 30 min for the initial attachment. The scaffolds were submerged after 30 min and incubated at 37 °C and 5% CO_2_, using a chondrocyte growth medium (Cell Applications Inc., USA). The media were changed every other day.

Prestoblue assay was used to measure the proliferation rate of chondrocytes on the Cartilage Portion. At certain time intervals (i.e. 4, 7, 14, 21 days), the media of the wells were replaced with 10% v/v prestoblue reagent. The plates were then incubated for 1.5 h at 37 °C and 5% CO_2_. The fluorescence intensity was measured at an excitation wavelength of 540 nm and emission wavelength of 590 nm. The same procedure was performed on day 1, after complete attachment of the cells to the scaffolds. The number of cells at each time point (N) to the initial number of the cells (N_0_) was calculated by dividing the corresponding intensity value by the absorbance value of the first day.

SEM was used to investigate cell attachment. Scaffold-cell complexes were washed using PBS and immersed in Karnovsky’s fixative for 1.5 h. The complexes were then submerged in 1% w/v osmium tetroxide for 1.5 h. Ethanol series (30, 50, 75, 95 and 100% v/v) were used to dehydrate the samples. The samples were then air-dried and sputter-coated with gold. SEM imaging was performed at accelerating voltages between 1 and 5 kV with different magnifications.

At the mentioned intervals considered for proliferation rate measurement (i.e. 7, 14, 21 days), scaffolds were fixed in formalin solution (10%, neutral buffered) for H&E staining. The cells on the scaffold were fixed overnight and stained using Eosin and Hematoxylin. The color was adjusted using 1% v/v acidic alcohol, ethanol and bluing agent, according to standard histology protocols. 3D confocal laser scanning microscopy (LEXT, Olympus) and fluorescence microscopy (Evos Fl, Life Technologies) were employed for monitoring cell growth on the scaffold and extracellular matrix (ECM) secretion by chondrocytes.

### Characterization of the film

#### Scanning electron microscopy (SEM)

SEM imaging was employed to investigate the morphology of porous PCL film (SEM, JEOL-JSM6510, Japan). Samples were sputter-coated with gold and imaging was carried out at accelerating voltage of 3 kV at various magnifications.

#### Mechanical properties and suture retention strength

Tensile strength of the films was examined using an Electromechanical Precision Universal Tester. The samples, fixed into a screw flat tensile grip, underwent the tensile test using a 5 kN load cell and crosshead speed of 1 mm/min. To investigate the effect the addition of PEG and the resulting porosity had on tensile strength, dense PCL films were also prepared using the solvent-casting method and tested under the same conditions.

To measure the suture retention strength, a steel wire of 0.15 mm diameter was used, by which the effects of suture materials on force–displacement curves is minimized. The steel wire was passed through a pinhole created in the Film to form a loop and fixed to the tensile testing machine, such that the distance from the grip was 10 mm. The other edge of the Film was fixed to the inferior screw flat tensile grip. The steel wire was pulled at a rate of 1 mm/min until the Film was completely torn.

#### Wettability and permeability

The wettability of the Film was evaluated through contact angle measurement^59^. A 5 µL drop of DI water was placed on either side of the samples, and the image was captured using a Dino-lite digital microscope camera. Each image was then processed to determine the contact angle.

**Figure 2:**
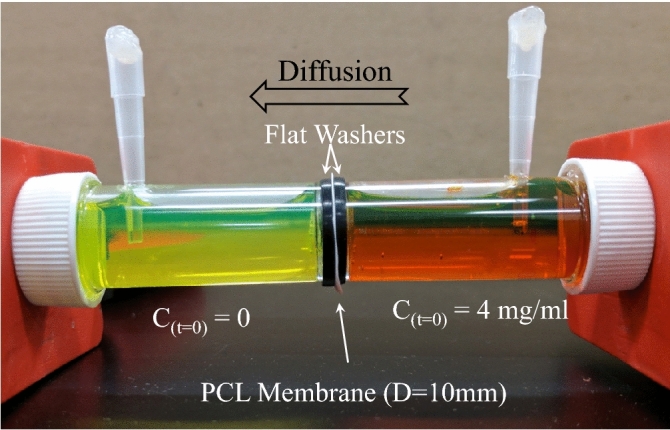
The experimental setup for monitoring the diffusion of the model probe, i.e. fluorescein sodium salt through the PCL Film.

Fluorescein sodium salt was selected as the model probe to quantify the permeability of the Film^60^. The Film (D = 10 mm) was fixed between two reservoirs filled with 4 mg/mL of solution or pure water. The entire system was fixed in a clamp and placed at 37 °C. At certain time intervals, 100 µL of the solution was taken out from the low concentration side, transferred to a 96-well plate and the intensity of fluorescence was documented at the excitation/emission wavelengths of 540/590 nm using a micro-plate reader. The diffusion kinetics were reported as the variation in the ratio of concentration at each specific time to the equilibrium concentration over time. Figure 2 displays the setup used for the experiment.

## Results and discussion

The majority of the inorganic component of natural bone is calcium phosphates, which makes this group of materials very populate in bone tissue engineering^61–63^. Among all calcium phosphates, HA (Ca_10_(PO4)_6_(OH)_2_) and TCP (Ca_3_(PO_4_)_2_) have been effective elements of many bone substitutes and scaffolds as they are able to effectively facilitate osteogenesis^64–67^. TCP is known as a bioceramic with higher degradability compared to other calcium phosphates^68–71^. The degradation of TCP-based composites can be regulated and slowed down by inclusion of HA. Moreover, HA is known as a bioceramic that can successfully interact with soft tissues (such as cartilage tissue in our case)^72^.

Although use of combined TCP and HA, compared to pure TCP and HA, is increasing due to their effectiveness and the tunable degradability^73–79^, their ratio in a composition should be selected based on the specific application. In SF 1, we have fully examined four ratios of TCP/HA in terms of their printability, degradability, rheological and mechanical properties and selected TCP/HA 80:20 paste as the most suitable ink to be used for printing the Bone Portion of our plug.

**Figure 3:**
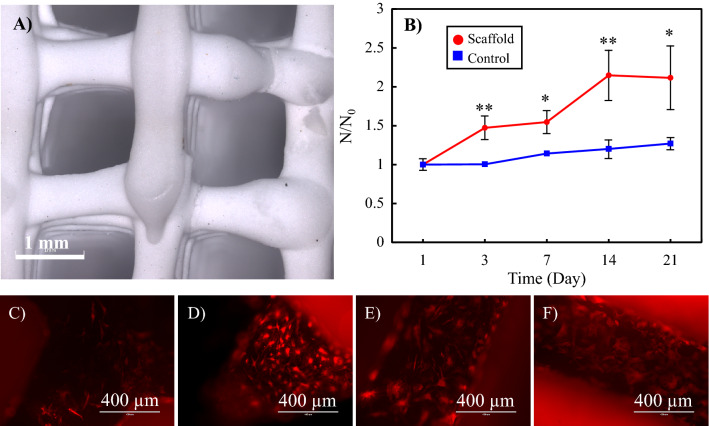
(**A**) 3D confocal laser scanning images of a 3D printed hard scaffold (TCP/HA 80:20) to be used for the Bone Portion of the Plug. (**B**)The proliferation rate of human osteoblasts on the scaffolds, as well as the standard cell culture plate (control group). *N0* initial number of the cells, *N* number of cells at each time point. Statistical significance *: p < 0.05, **: p < 0.01, p-values in Day 3: 0.006, Day 7: 0.011, Day 14: 0.006 and Day 21: 0.023. (**C**–**F**) The fluorescence images of the attached osteoblasts on the scaffolds after (**C**) 3, (**D**) 7, (**E**) 14 and (**F**) 21 days.

The results of the TCP/HA 80:20 paste rheologic analysis (SF 1, Fig. S2B1,B2) indicates that this ink appears to have a yield flow stress. The shear stress-shear rate curves suggest the Herschel-Bulkley type behavior. Temperature change does not have a significant effect on the rheological properties of the paste. The morphology of the printed and sintered scaffolds is shown in Fig. 3A. The surface roughness of the scaffolds was also measured using the 3D confocal laser scanning microscope. The arithmetic mean height (S_a_) and root mean square height (S_q_) were found to be 1.73 ± 0.47 µm and 2.23 ± 0.57 µm, respectively.

The porosity of the scaffolds was 25.5 ± 3.8%. The mechanical testing was performed on the scaffolds made with the structure needed in the plug (i.e., 3D printed cylinder with the diameter of 20 mm, height of 7.5 mm and pore size of 850 µm). The values obtained for Young modulus (E), compressive strength (σ_B_) and fracture strain (ε_R_), by four repetitions in four separate samples, include E = 218.12 ± 19.10 MPa, σ_B_ = 39.08 ± 11.74 MPa and ε_R_ = 16.40 ± 5.66% (n = 4). Although the fracture strain of the scaffold is relatively small, the Young modulus and compressive strength are well above the average values reported for trabecular bone^80^.

The proliferation rate of osteoblasts on the scaffolds, as well as in the control group (cell culture plate), is shown in Fig. 3B. The results are reported as the variation of N/N_0_ over time, which is the ratio of the number of cells at each time point (N) to the initial number of the cells (N_0_). The ratio can be obtained by dividing the corresponding intensity values.

As observed, except for the starting point, the number of cells on the scaffold was higher than the control group at all other time points. Furthermore, the cells in the scaffold had improved growth and proliferation compared to the control group.

**Figure 4:**
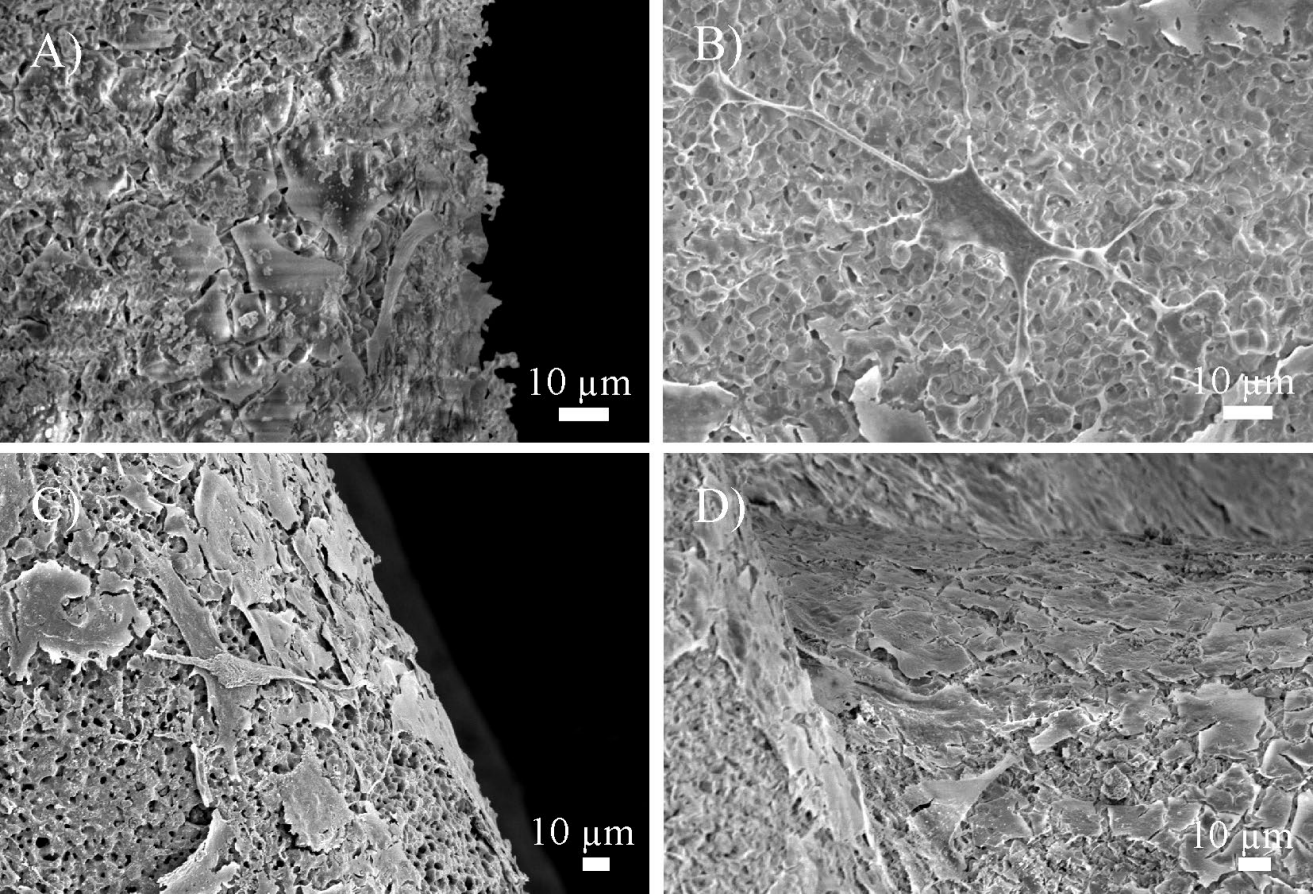
SEM images of the attached osteoblasts on the scaffolds of the Bone Portion after (**A**) 3, (**B**) 7, (**C**) 14 and (**D**) 21 days.

Figures 3C–F and 4 display the fluorescence and SEM images of the attached osteoblasts on the scaffolds, respectively. The growing osteoblasts, in the form of adherent plexus, can be seen in the pores of the scaffolds. The cytoskeletal projections, lamellipodia and filopodia of the cells anchored visibly on the surface features, which indicates the bioactive properties of the scaffolds.

The composition of the scaffold used for cartilage regeneration can have a significant impact on cell behavior. Cartilage Portion in our plug is made of gelatin/elastin/sodium-hyaluronate. Gelatin-based hydrogels have been reported to promote the chondrogenic differentiation of mesenchymal stem cells^81^. The up-regulation of chondrogenic markers—such as SOX9, collagen type II, aggregan and versican—has been shown for gelatin-based scaffolds^82^. Addition of hyaluronic acid to gelatin-based scaffolds may even improve their chondrogenic activity. Chondrocyte proliferation, adhesive activity and new hyaluronic acid production have been shown to significantly increase in hyaluronic acid-treated gelatin scaffold, compared to non-treated groups. Furthermore, higher quality of hyaline-like extracellular matrix production and a higher filling of extracellular matrix in the pore of scaffold have been demonstrated for hyaluronic acid-treated gelatin scaffold via immunohistochemistry and SEM, respectively^83^. Such an effect can be pursued in the mechanism of hyaluronic acid interaction with chondrocytes. Chondrocytes express the glycoprotein CD44 on their cell surface; this has the capacity to function as a hyaluronic acid receptor and, therefore, may be involved in biochemical interactions with chondrocytes^84,85^. Hyaluronic acid binds to CD44 and acts on intracellular signal transduction via protein kinase C as a result of the transmission of the bound hyaluronic acid to the cytoplasm. Hyaluronic acid increases the synthesis of proteoglycans and collagen by chondrocytes^86^. On the other hand, incorporation of elastin into the formulation might also influence the chondrogenic activity. According to the study conducted by Betre et al.^87^, elastin-like polypeptide can promote chondrogenesis for human adipose-derived adult stem cells in the absence of exogenous TGF-β1 and dexamethasone. Significant increases in sulfated glycosaminoglycan (up to 100%) and collagen contents (up to 420%) have been observed by these investigators. Immunolabeling confirms that the matrix formed consists mainly of type II, and not type I, collagen^87^.

**Figure 5:**
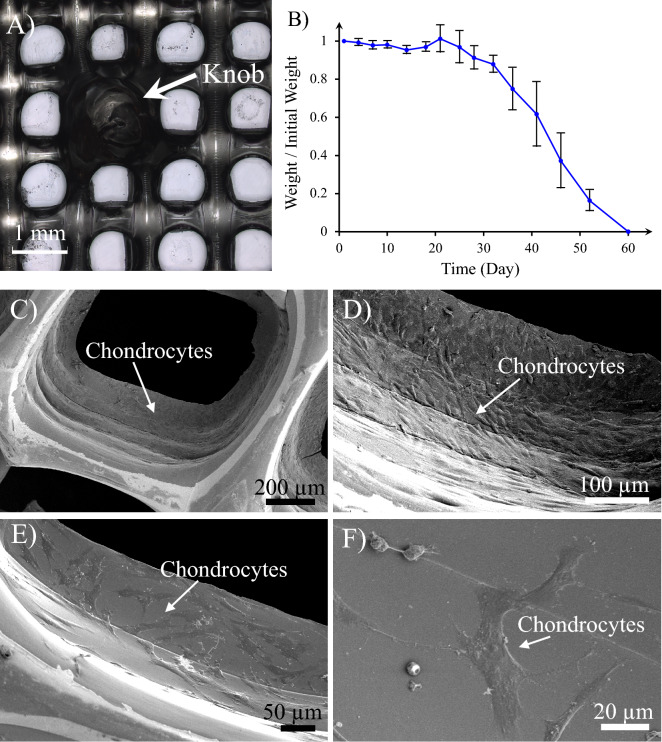
(**A**) 3D printed construct of the Cartilage Portion (gelatin/elastin/sodium hyaluronate soft membrane). The knobs on the 3D printed membrane are constructed to mechanically interlock the Cartilage Portion to the Bone one. (**B**) Degradation kinetics of the Cartilage Portion—the weight of the sample at each time point has been normalized to the initial weight of the sample and exploited as an indicator for degradation magnitude. (**C**–**F**) Chondrocyte attachment on the gelatin/elastin/sodium-hyaluronate construct one day after seeding. The magnification of SEM images increases from (**C**) to (**F**).

The morphology of the printed gelatin/elastin/sodium hyaluronate scaffold for the Cartilage Portion of the plug is illustrated in Fig. 5A. A knob that has been designed for interlocking the Bone and Cartilage Portions can be seen in the image. The average pore size was found to be 892 ± 62 µm. The degradation rate of the Cartilage Portion is demonstrated in Fig. 5B. The scaffold weight remained almost constant in the first 30 days, implying the stability of the constructs in this timeframe, but decreased drastically between 30 and 60 days. After 60 days, the scaffolds completely collapsed and disappeared.

The mechanical properties of similar membranes made of same ratio of gelatin, elastin and sodium hyaluronate have been quantitatively characterized in our previously reported studies for membranes with 150–200 µm thickness in terms of suture retention strength (20.4 ± 2.03 g), Young’s modulus (170.2 ± 36.1 kPa–1.95 ± 0.55 Mpa and ultimate strength (95.4 ± 10.1 kPa–1.15 ± 0.33 Mpa)^88,89^. The appropriate suturability of the membrane allows the possible assembly of the Cartilage Portion with the Bone Portion using sutures. The appropriate mechanical properties of the membrane allows its appropriate surgical handling, which was assessed by a knee surgeon who did not have prior knowledge regarding the make and composition of the Cartilage Portion to avoid the bias in his evaluation. More specifically, the surgeon evaluated the easiness of manipulation and cutting with surgical forceps and scissor, suturability, and firmness in terms of how the Cartilage Portion can lie on the Bone Portion.

By also assured by the objectivity of the surgical handling assessment was ensured by assigning three different surgeons with subspecialty in Cornea and Ocular Surface to conduct the operations. Surgeons did not have any prior knowledge about the make and composition of the scaffolds to mitigate the bias in their evaluations. The assessment comprised of suturability, ease of manipulation and cutting with surgical forceps and scissor, and rigidity in terms of how the scaffold lied on the tissue.

As shown in Fig. 5C–F, after 1 day of cell seeding, chondrocytes attach perfectly to the surface of the gelatin/elastin/sodium-hyaluronate construct.

**Figure 6:**
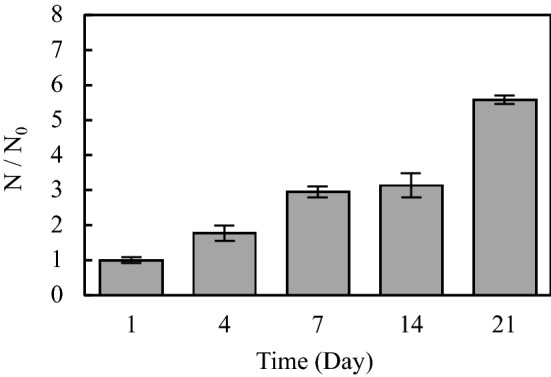
The proliferation rate of normal human chondrocytes on the cartilage (gelatin/elastin/sodium hyaluronate) constructs. *N* number of cells at each time point, *N*_*0*_ initial number of the cells.

The proliferation rate of normal human chondrocytes on the scaffolds is illustrated in Fig. 6. The results are reported as the variation of N/N_0_ over time, which is the ratio of the number of cells at each time point (N) to the initial number of the cells (N_0_). The ratio can be obtained by dividing the corresponding intensity values.

The number of cells on the scaffold persistently increase from day 1 to day 21. After 21 days, the number of cells was more than five times that of the initial number seeded on the first day.

**Figure 7:**
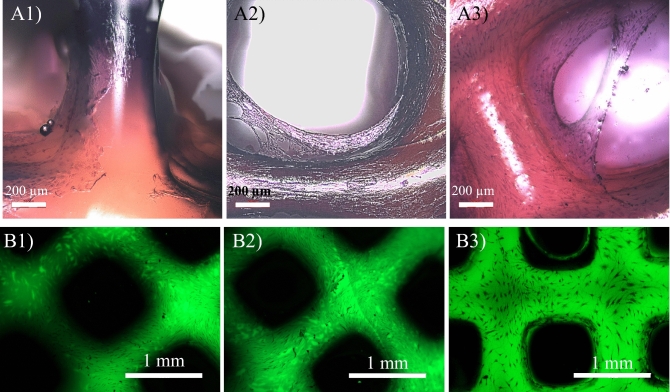
(**A1**–**A3**) Chondrocyte attachment, growth and ECM secretion on gelatin/elastin/sodium hyaluronate scaffolds after (**A1**) 7, (**A2**) 14 and (**A3)** 21 days post-seeding. The samples were stained with Eosin/Hematoxylin using standard histology protocols and the images were taken using a 3D confocal laser scanning microscope (label-free). (**B1**–**B3**) Fluorescence images of the chondrocytes growing on the gelatin/elastin/sodium-hyaluronate construct after (**B1**) 7, (**B2**) 14 and (**B3**) 21 days.

Both 3D confocal laser scanning microscopy (label-free) and fluorescence microscopy confirm the attachment, growth, proliferation and ECM secretion over 21 days, as shown in Fig. 7.

“Fibroblastic” morphology of chondrocytes was observed on our scaffold. This is in agreement with the study by Awad et al.^90^, in which the authors investigated the chondrogenic differentiation of adipose-derived adult stem cells in scaffolds made of gelatin, alginate and agarose. In this study, the cells in the agarose and alginate scaffolds presented a spherical morphology that persevered during the period of culturing. However, cells grown in scaffolds made of gelatin showed a distinct “fibroblastic” morphology, proliferating and becoming confluent with distinguished cell-to-cell contact. In general, Awad et al. did not find any substantial differences in chondrogenic differentiation capability of scaffolds with different materials, despite alterations in the cell morphology^90^.

A high proliferation rate of chondrocytes was observed in this study. The chondrocyte cell cycle varies according to species and location. The doubling time of human chondrocytes in monolayer cultures ranges between 1.7 and 3.5 days, according to various studies^31,91,92^. Our scaffold maintained the typical proliferative activity of the chondrocytes.

**Figure 8:**
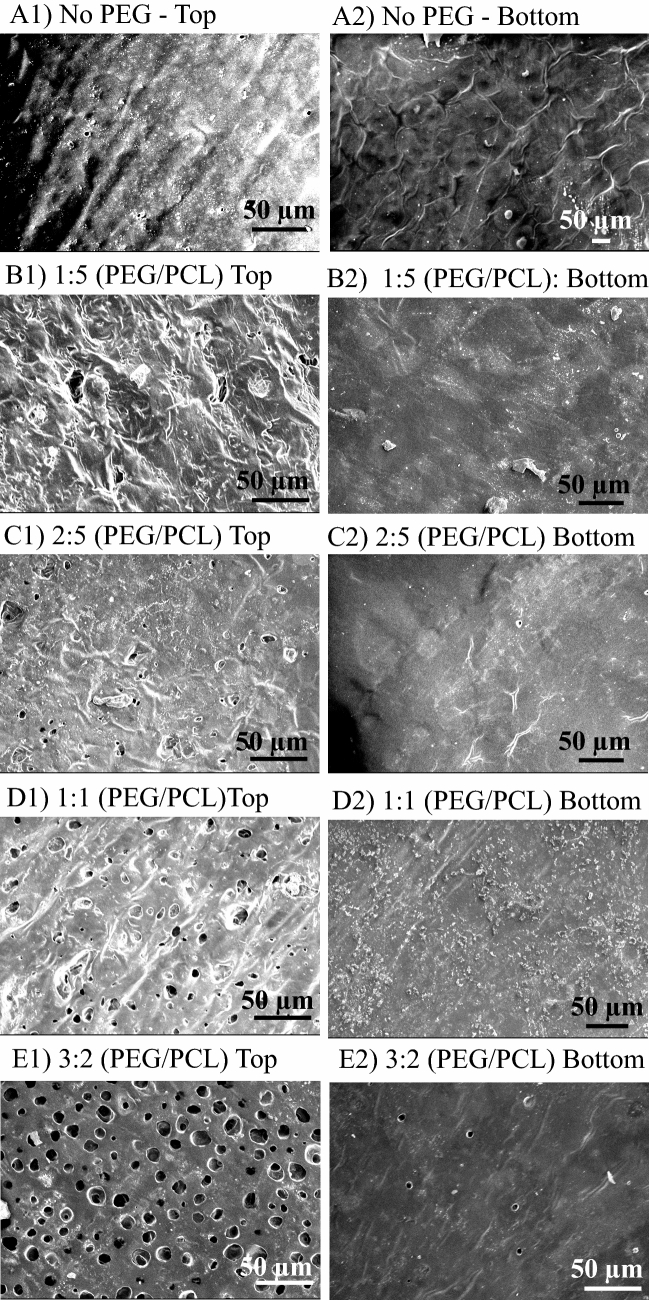
SEM images of both sides (top and bottom) of PCL membranes prepared by addition of different amount of PEG. PEG/PCL w/w ratio is 0 (**A**), 1:5 (**B**), 2:5 (**C**), 1:1 (**D**) and 3:2 (**E**). Only the ratio of 3:2 PEG/PCL formed pores in both sides, which is selected to be used as the Film in the knee plug. The average size of the formed pores in the Film is 10 µm.

For optimization of the permeable PCL membrane to be used as the third component of the knee plug, Film, PCL constructs with various amount of PEG were prepared. Figure 8 illustrates the SEM images of both sides of PCL membrane (top and bottom) made by addition of various amounts of PEG. As shown in this figure, addition of either none or a very small amount of PEG, with the PEG/PCL w/w ratio of 1:5 and 2:5, resulted in none or a very small number of pores per unit of surface area (Fig. 8A–C). Through employing 1:1 PEG/PCL w/w ratio, more pores were observed on one side (Fig. 8D1), however, water is unable to pass through the membrane, even when a high vacuum was applied to the other side as a driving force. A possible explanation for this is the formation of no pore at the other side (Fig. 8D2). While there were adequate pores in one side, they seemed to be close-ended, as the transportation of water molecules was still not possible. However, increasing the ratio to 3:2 w/w resulted in the formation of some pores on the other side, through which the water molecules could instantly permeate by applying a vacuum on our observation. We can see the pore on both sides of membrane in Fig. 8E1,E2. Therefore, this sample was selected as the optimal Film and used for further characterization. As shown in Fig. 8E, the pores on the optimum porous PCL Film were found to be approximately 10 µm. Such pore size allows diffusion of nutrients, like glucose, but prevents cell migration.

Table 3 represents the mechanical properties and suture retention strength of the prepared Films compared to solid PCL membrane fabricated with addition of no PEG. The porous PCL Film was found to have lower modulus and ultimate strength, but higher elongation at break. Having a porous structure, as well as the plasticizing effect of the residual PEG, might account for lower stiffness and strength, along with more flexibility. The suture retention strength was 9.04 ± 0.92 N for the porous PCL Film, which was about half of the corresponding value for solid membrane, but was still strong enough to be sutured to the surrounding tissues or scaffolds as a means of engagement.

**Table 3:** The mechanical properties and suture retention strength of porous PCL Film compared to a solid PCL membrane.

Parameter	Solid PCL membrane	Optimized porous PCL film
Young modulus (MPa)	112.02 ± 3.62	16.91 ± 2.75
Ultimate strength (MPa)	12.96 ± 0.99	4.91 ± 1.36
Elongation at break %	566.83 ± 71.07	973.02 ± 15.35
Suture retention strength (N)	18.04 ± 5.28	9.04 ± 0.92

**Figure 9:**
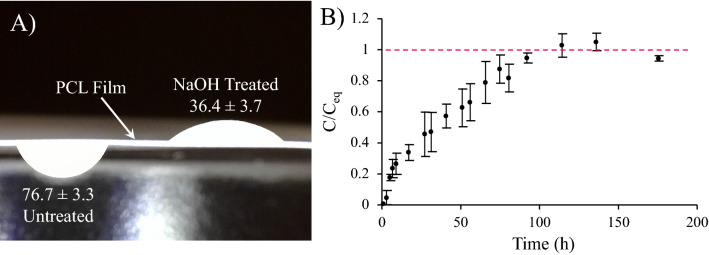
(**A**) The effect of treatment with NaOH on the wettability and contact angle of the PCL Film. (**B**) The diffusion kinetics of the model probe (i.e. fluorescein sodium salt) through the Film (*C* concentration, *C*_*eq*_ concentration at equilibrium).

The treatment of the surface of the Film with NaOH resulted in improved wettability, as displayed in Fig. 9A. Floating the Film on NaOH solution resulted in a dramatic decrease in the contact angle from 76.7 ± 3.3° to 36.4 ± 3.7°.

Wettability has been reported to affect protein adsorption and cell adhesion^93,94^. The number of adhered human umbilical vein endothelial cells (HUVECs) and HeLa cells has been shown to reach a maximum on the surfaces with a water contact angle of 40° and 50°, respectively, and decreases with further increase of water contact angle up to 60–70°^93^. Having the Film on top of the plug is supposed to help retaining the cells within the plug. Having a Film with one hydrophilic (the side facing the Cartilage Portion) and one hydrophobic side, facilitates such retention as cells avoid the hydrophobic sides and attach the hydrophilic side.

The Film was also found to be permeable to the model probe, as shown in Fig. 9B. The diffusion kinetics were faster at the beginning due to a relatively large concentration gradient, but they then decreased over time and eventually reached a plateau after about 100 h. Figure 9B illustrates the variation of the model probe concentration in the receiver reservoir normalized to the equilibrium concentration.

Effective permeation of extracellular fluids and nutrients has been listed as one of the important features that a biodegradable polymeric membrane should have to be used for tissue regeneration^95,96^. The permeability of the PCL Film allows the nutrient delivery and waste removal needed for the healthy growth of the cartilage and bone cells. Moreover, considering the size of chondrocytes which is approximately 20 µm^97^, the small pore size of the Film (10 µm) prevents the possible migration of cells outside of the plug.

The knee plug fabricated in this paper can support both bone and cartilage growth, while keeping favourable cell differentiation between osteoblasts and chondrocytes. One of the functions of this tri-component knee plug is simplifying the surgical procedure employed for the joint repair. Bone and Cartilage Portions may be seeded with cells and then inserted into the designated area of the body jointly as a unit or separately. They can also be inserted into the joint without cells, and then cells seeded into the relevant components after implantation of each component.

This design considers appropriate accommodation of both osteoblasts and chondrocytes involved in the ACI surgery. Having the large pore size in this design facilitates the nutrient delivery and waste removal and thus assists the cell growth, while resolving the issue of cell retention—a challenge of ACI method.

## Conclusion

In this paper we presented a new knee plug consisting of three components—namely Bone Portion, Cartilage Portion and Film—to be used in the 3rd generation of ACI surgeries with the following advantages: providing a suitable platform for growth of the relevant cells in the area of knee cartilage, simplifying the surgery, control over the pore size and structure, stitchability, use of synthetic materials, possible low cost and less morbidity of other parts of the body during the surgery. The components of this plug are in the size of the tissue normally removed for ACI surgeries in human and can be engaged to each other. The in vitro analyses revealed that normal human chondrocytes can perfectly attach to this plug with appropriate proliferation rate.

## Supplementary information

Supplementary Information.
