# Supplementary material for: A tri-component knee plug for the 3rd generation of autologous chondrocyte implantation
Source: Sci Rep. 2020 Oct 12;10:17048. doi: 10.1038/s41598-020-73863-x (PMC7550599; doi:10.1038/s41598-020-73863-x)
Supplement: Supplementary file 1 — Supplementary Information. [file 41598_2020_73863_MOESM1_ESM.docx]

**A Tri-Component Knee Plug for the 3^rd^ Generation of Autologous Chondrocyte Implantation**

**Supplementary File 1: Optimizing the Paste to Print the Bone Portion**

Lobat Tayebi*^1, 2^*, Zhanfeng Cui*^1^,* Hua Ye*^1,*^*

*^1^*Institute of Biomedical Engineering, Department of Engineering Science, University of Oxford, Oxford OX3 7DQ, UK

*^2^*Marquette University School of Dentistry, Milwaukee, WI 53233, US

*^*^*Corresponding Author: Email: [hua.ye@eng.ox.ac.uk](mailto:hua.ye@eng.ox.ac.uk), Phone +44 (0)1865 617689

To select the most appropriate paste for printing the Bone Portion of the knee plug, this Supplementary Material (SM) aims to examine the four different compositions of pastes in which the β-tricalcium phosphate (TCP) and hydroxyapatite (HA) have the ratios as follows:

Formulation 1) TCP/HA 100:0

Formulation 2) TCP/HA 80:20

Formulation 3) TCP/HA 70:30

Formulation 4) TCP/HA 50:50

**1. Materials and Method**

**1.1. Preparation of the Pastes**

Starting from formulation 1 (TCP/HA 100:0) the paste was made by mixing 15 g of TCP with 5.75 ml water, 0.5 g sodium tripolyphospahte (TPP, Alfa Aesar) and 75 mg carboxymethyl-cellulose (CMC, Alfa Aesar). This formulation was previously developed as a printable TCP scaffold [1]. We hypothesize that incorporation of HA can improve the mechanical properties. Thus, since sufficient mechanical properties are crucial in bone scaffolds, we replaced some TCP by HA in formulations 2, 3 and 4. For this purpose, the total weight of ceramics (15 g) was held constant but divided between TCP and HA based on their defined ratio in each formulation. More specifically, the amount of TCP and HA are as follows:

Formulation 1) 15 g TCP, Formulation 2) 12 g TCP + 3 g HA, Formulation 3) 10.5 g TCP + 4.5 g HA, and Formulation 4) 7.5 g TCP + 7.5 g HA.

All formulations were homogenized at 2000 rpm for 2 minutes (min) using a centrifugal mixer (Thinky, USA).

**1.2. Rheological Analysis**

To perform the rheological analysis of each formulation, the viscosity and applied stress were measured as a function of shear rate at different temperatures. For this purpose, a shear rheometer (Kinexus, Malvern, UK) with a stainless steel parallel-plate geometry of 20 mm in diameter and a Peltier temperature control was used. The viscometry of the samples was conducted, applying a gap distance of 0.5 mm at temperatures increasing in 2°C increments, ranging from 20°C up to 26°C. The shear rate varied logarithmically in ramp mode from 0 to 50 s^-1^ and then back to 0 s^-1^.

## 1.3. 3D Printing and Sintering

## The scaffolds were fabricated using a 3D-BIOPLOTTER system (EnvisionTEC, Germany). By applying the optimized parameters (air pressure of 1-1.5 bar at a speed of 4-7 mm/s), the pastes were extruded from a cartridge through a plotting needle with a diameter of 250 µm (Nordson, USA). The printer head deposited strands of the pastes in a layer-by-layer fashion on the platform, forming a disc with 6 mm diameter and 3 mm thickness. Scaffolds were air-dried overnight and then sintered. For sintering, temperature of the furnace was raised to 600°C at the constant rate of 3°C/min, held for 1 hour (h) in 600°C, then raised from 600°C to 1100°C at a rate of 5°C/min and remained at 1100°C for 4 h.

**1.4. Mechanical Testing and Degradation Analysis**

The Young modulus and compressive strength of the scaffolds were measured using a mechanical testing machine (Shimadzu, Japan), with a 5kN load cell and a cross-head speed of 1.0 mm/min.

To evaluate the degradation behavior of the samples, they were immersed in PBS for 12 weeks. Since their weight were not changed noticeably during this time (especially for the samples with higher percentage of HA), we have evaluated their degradation behavior by measuring their mechanical properties. At certain times, samples were taken out, and their Young modulus and compressive strength were measured using a 5 kN load cell and crosshead speed of 1 mm/min.

**2. Result and Discussion**

All four formulations were 3D printed in the form of disc with a diameter of 6 mm and thickness of 3 mm. Figure S1 presents their structures. During the printing procedure, it was determined that formulations 1 and 2 are the most robust pastes for printing. More specifically, only about 5% of the constructs made with these pastes had uneven strands. This number is about 30% for formulation 3, due to the clogging of the dispenser. Printing of formulation 4 failed very frequently; 7 out of 10 constructs (70%) made with this formulation were composed of uneven strands due to frequent blockage during the printing procedure.

Please note that HA particles were sieved using a 45-micron mesh prior to be mixed in the formulation. Considering the use of a much larger nozzle size (250 µm diameter) for the printing procedure, the frequent clogging of formulations 3 and 4 cannot be attributed to the HA particle size, but might mostly be relevant to the fact that HA particles were prone to agglomeration. Thus, we concluded that their amount in the formulations must be limited and not exceed the 20% of the ceramic content.

Figure S1: Microscopic images of the 3D printed scaffolds with the TCP/HA ratio of: (A) 100:0 [formulation 1], (B) 80:20 [formulation 2], (C) 70:30 [formulation 3] and (D) 50:50 [formulation 4].

The rheologic properties of these four pastes may also help explaining the encountered difficulties in the printability of formulations 3 and 4. More specifically, analyzing Figure S2, one may realize two considerations that aid in understanding the behavior of the pastes during the printing procedure:

1) Formulations 3 and 4 are more temperature sensitive compared to formulations 1 and 2 at lower temperatures. It is especially important because lower temperatures (20-22°C) are more favorable for printing due to reduced evaporation which in turn improves consistency. The room temperature, in which the printing was performed, was also around 20-22°C. Formulation 2 appears to be the least sensitive paste to temperature which makes it reliable during the printing procedure.

2) For most of the compositions/situations, the viscosity is increasing with increasing HA. This can be generalized to all the ratios at low temperatures (20°C). The viscosity values of formulations 3 and 4 are very high, which make them difficult to be handled for smooth 3D printing.

Figure S2: Shear stress versus shear rate and shear viscosity versus shear rate at various temperatures for the pates with the TCP/HA ratio of: (A1,A2) 100:0 [formulation 1], (B1,B2) 80:20 [formulation 2], (C1,C2) 70:30 [formulation 3] and (D1,D2) 50:50 [formulation 4].

Please note that the shear stress versus shear rate is typically ascending and the fluctuating seen in formulation 4 at some temperatures can be considered as an artifact, or irregular behavior of this formulation.

Considering the above facts about the printability and rheological properties of all formulations, we realized that formulations 3 and 4 were definitely not suitable for printing. We further studied the formulations by analyzing the mechanical and degradation properties of the scaffolds made by these compositions.

Figure S3: Mechanical properties (Young modulus and compressive strength) and degradation of the printed scaffolds using the ink composed of TCP/HA ratio of: 1) 100:0, 2) 80:20, 3) 70:30 and 4) 50:50 were measured immediately after preparation and storage in SBF for 4, 8 and 12 weeks. The values of Young modulus and compressive strength are obtained after three repetitions on three separate samples. All scaffolds were in the shape of a disc with 6 mm diameter, 3 mm thickness, porosity of 41.9 ± 2.5% and the inner structure as shown in Figure S2.

The mechanical properties of the printed scaffolds were measured immediately after preparation and storage in PBS for 4, 8 and 12 weeks. Young modulus and compressive strength of the scaffolds are presented in Figure S3. The values of Young modulus and compressive strength are extracted after three replications on three separate samples.

In general, the values of Young modulus and compressive strength increased by increasing the HA content in the compositions. Also, the mechanical properties declined by increasing storage time in PBS, which is indicative of scaffold degradation. However, many of these changes in the values of Young modulus and compressive strength from one composition to another and from one week to another are not significant, and thus, not conclusive in determining the best formulation. However, the two considerations below, along with the previously mentioned points about the printability and rheological properties of TCP/HA 80:20, are convincing that formulation 2 can be an optimized choice:

1) Young modulus is significantly increased in formulation 2 compared to formulation 1 by addition of 20% HA—from TCP/HA 100:0 to 80:20—in most of the weeks. However, this increase is not significant when the addition of HA continued to 30 and 50% in formulations 3 and 4. Thus, having 20% HA seems to be sufficient, and it is not necessary to increase the amount of HA further, as it does not significantly improve the mechanical properties after 20%, at least in the printed samples under the mentioned circumstances.

2) As shown in Figure S3, although scaffolds with all formulations demonstrate a continuous decrease in mechanical properties after storage in PBS solution over time, this decrease for formulations 3 and 4 is very slow, which indicates the exceedingly slow degradation property of them. During the experimental procedure, no noticeable change in the morphology of these scaffolds was observed after period of 12 weeks. This result is in agreement with the reported property of HA regarding the slow degradation of this material (in the order of 10^-3^ g per two weeks storage in PBS) [2, 3]. Since we are seeking a degradable scaffold in which the degraded materials can be replaced by the growing cells, such a slow degradation is not favourable.

Considering the two above points, which favour formulation 2 over formulation 1, along with the facts about the difficulty in printability and rheological properties of formulation 3 and 4, formulation 2 is selected as the optimized paste to be used in fabrication of the scaffold for the Bone Portion.

Note that we observed a slight increase in the Young modulus and compressive strength of the scaffold made specifically for the Bone Portion of the plug (cylinder with the diameter of 20 mm, height of 6-8 mm and pore size of 850 µm), compared to the scaffold made for studying the various formulation in this study (disc with 6 mm diameter, 3 mm thickness and pore size of 250 µm). This difference can be attributed to the different distance between strands as well as use of different nozzles during printing (250 µm versus 410 µm), which resulted in different porosities. More specifically, the porosity of the discs made in this SM was 41.9 ± 2.5%, while the porosity of the scaffolds made for the Bone Portion was 25.5 ± 3.8%.

**3. Conclusion**

To find the most appropriate scaffold for the Bone Portion of the plug, four formulations of pastes with the TCP/HA ratio of : A)100:0, B) 80:20, C)70:30 and D) 50:50 were examined. After studying of the printability, rheological, mechanical and degradation properties of all formulations, the paste with the TCP/HA ratio of 80:20 was ultimately selected as the most optimized paste for 3D printing of the Bone Portion.

**References**

[1] F. Fahimipour, E. Dashtimoghadam, M. Rasoulianboroujeni, M. Yazdimamaghani, K. Khoshroo, M. Tahriri, A. Yadegari, J.A. Gonzalez, D. Vashaee, D.C. Lobner, Collagenous matrix supported by a 3D-printed scaffold for osteogenic differentiation of dental pulp cells, Dental Materials 34(2) (2018) 209-220.

[2] H. Wang, J.K. Lee, A. Moursi, J.J. Lannutti, Ca/P ratio effects on the degradation of hydroxyapatite in vitro, Journal of Biomedical Materials Research Part A: An Official Journal of The Society for Biomaterials, The Japanese Society for Biomaterials, and The Australian Society for Biomaterials and the Korean Society for Biomaterials 67(2) (2003) 599-608.

[3] H. Wang, Hydroxyapatite degradation and biocompatibility, The Ohio State University, 2004.
